# Unusual Migration of a Gastric Band 15 Years Post-surgery: Laparoscopic Management of a Challenging Case

**DOI:** 10.7759/cureus.87533

**Published:** 2025-07-08

**Authors:** Ioanna Verzoviti, Katerina Delladetsima, Dimitrios Keramidaris

**Affiliations:** 1 Department of General Surgery, 417 Army Shared Fund Hospital, Athens, GRC

**Keywords:** adjustable gastric band (agb), band erosion, band migration, bariatric surgery, laparoscopic removal

## Abstract

The laparoscopic adjustable gastric band surgery (LAGB) is a surgical technique that was widely used in the past for the treatment of morbid obesity. Nowadays, it is on the decline due to its moderate results in weight loss, coupled with a significant risk of complications. Among others, band erosion and migration are relatively common and require gastric band removal. These conditions might be difficult to manage, as there is also a high possibility of post-surgical complications. Herein we present the case of a 46-year-old woman with port protrusion and erosion, as well as migration of the band in an atypical position inside the abdomen, occurring 15 years after a gastric band procedure, and its successful management.

## Introduction

Morbid obesity is a worldwide epidemic and, over time, numerous types of anti-obesity procedures have been described. One of them is the laparoscopic adjustable gastric band surgery (LAGB), a reversible type of bariatric surgery procedure. In this procedure, an adjustable silicone band is placed around the upper part of the stomach that reduces gastric capacity and decelerates the passage of food through the stomach [1,2]. The use of this anti-obesity technique tends to be abandoned due to its relatively modest amount of expected weight loss. Additionally, it carries high rates of several complications such as gastric ischemia, band erosion, and band migration through the gastric lumen that could respectively result in gastric obstruction, bowel obstruction, bowel perforation, esophagogastric fistula, or even discitis and osteomyelitis [3-7]. The management of these conditions might be challenging, with a high risk of postoperative complications as well. The symptoms of migration are variable but often include epigastric pain, nausea, vomiting, acid reflux, dysphagia, reservoir infection, or no restriction and weight loss despite aggressive band inflation [1]. However, it should be mentioned that gastric band migration might also be asymptomatic [8]. We describe a case of a 46-year-old woman with a band migration occurring 15 years after a laparoscopic band procedure and its successful management.

## Case presentation

**Figure 1:**
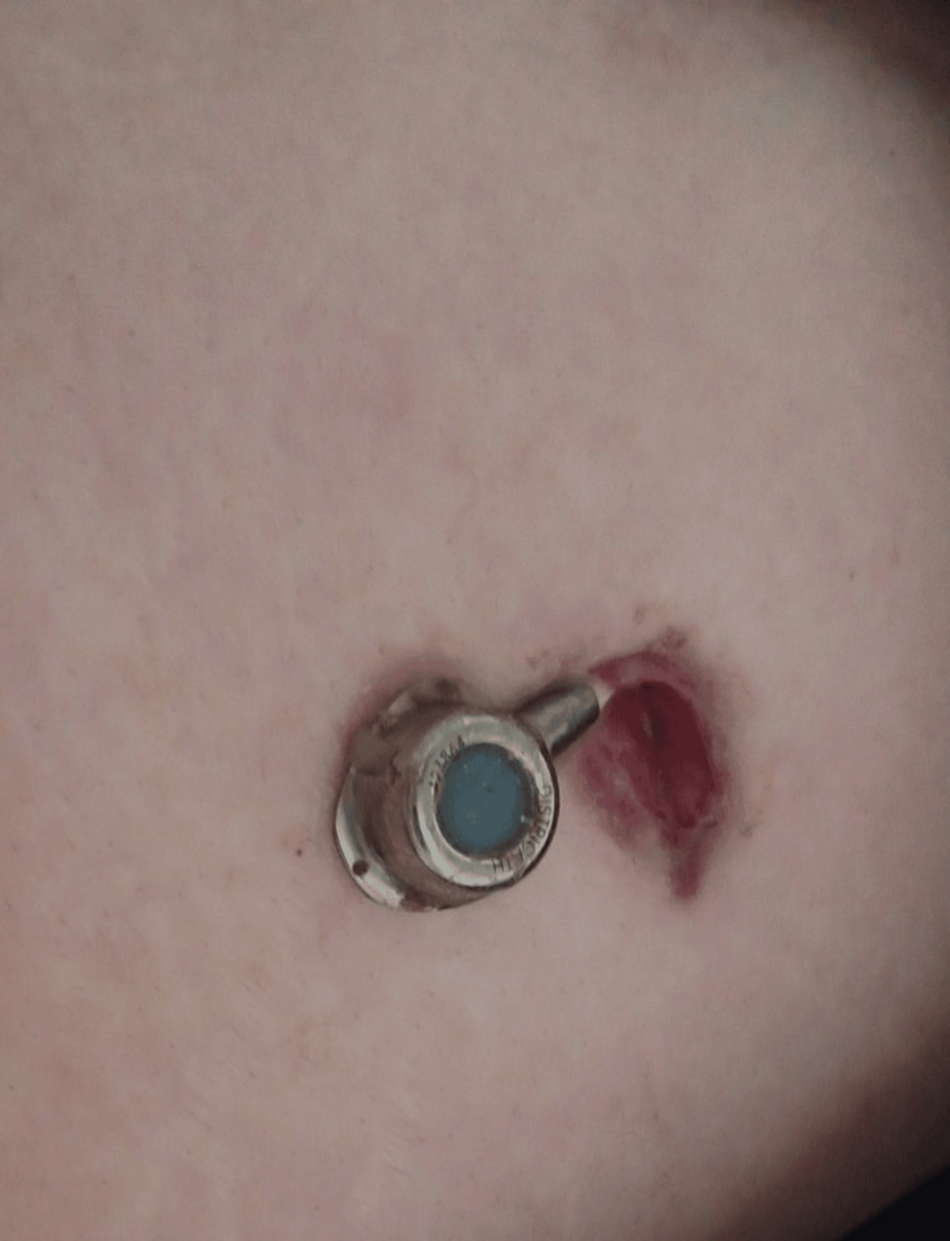
Port erosion of the abdominal wall and skin protrusion

A 46-year-old woman was referred to our department in order to remove the gastric band that was placed 15 years ago. Μedical history only revealed a surgical excision of an echinococcus liver cyst at the age of 14. The gastric band had to be removed due to port erosion of the abdominal wall and skin protrusion (Figure 1).

Moreover, the patient was not followed by any bariatric center in the past years and experienced only negligible weight loss after the gastric band placement. No other symptoms were reported. The preoperative evaluation was normal, and blood tests revealed only elevated C-reactive protein baseline (CRP-B) levels (13.1 mg/dL) (Table 1).

**Table 1:** Laboratory findings SGOT (AST): serum glutamic-oxaloacetic transaminase (aspartate aminotransferase); SGPT (ALT): serum glutamic-pyruvic transaminase (alanine aminotransferase)

Hematology test	Results	Unit	Normal range
White blood cells (WBC)	8.4	K/μL	4.0 - 10.0
Neutrophils %	56.9	%	40.0 - 75.0
Lymphocytes %	29.1	%	20.0 - 45.0
Monocytes%	8.4	%	2.0 - 10.0
Eosinophils %	5.2	%	0.0 - 6.0
Basophils %	0.4	%	0.0 - 2.0
Neutrophils (absolute number)	4.8	K/μL	2.0 - 7.5
Lymphocytes (absolute number)	2.4	K/μL	1.0 - 4.0
Monocytes (absolute number)	0.7	K/μL	0.2 - 1.0
Eosinophils (absolute number)	0.5	K/μL	0.0 - 0.5
Basophils (absolute number)	0.03	K/μL	0.0 - 0.2
Hemoglobin	13.9	g/dL	12.0 - 16.0
Hematocrit	40.7	%	37.0 - 46.0
Red blood cells (RBC)	4.53	M/μL	4.0 - 5.5
Mean corpuscular hemoglobin (MCH)	30.7	pg	26.0 - 34.0
Mean corpuscular hemoglobin concentration (MCHC)	34.2	g/dL	31.0 - 37.0
Mean corpuscular volume (MCV)	89.7	fL	79.0 - 100.0
Red cell distribution width – coefficient of variation (RDW-CV)	12.7	%	11.0 - 14.0
Platelets (PLT)	374	K/μL	140 - 400
Mean platelet volume (MPV)	10.2	fL	7.0 -11.0
Platecrit (PCT)	0.36	%	0.15 - 0.40
Nucleated red blood cells (NRBC) %	0.0	%	0.0
Biochemistry test	-	-	-
CRP-B (C-reactive protein-baseline)	13.1	mg/dL	< 0.5
Direct bilirubin	0.29	mg/dL	0.20 - 1.10
Total bilirubin	0.86	mg/dL	2.0 - 1.10
Uric acid	4.6	mg/dL	2.0 - 7.0
Creatinine	0.86	mg/dL	0.70 - 1.4
Urea	32	mg/dL	20 - 50
Glucose	102	mg/dL	70 -110
Sodium (Na+)	138	mmol/L	136 - 145
Potassium (K+)	4.0	mmol/L	3.5 - 5.0
SGOT (AST)	27	IU/L	5.0 -40.0
SGPT (ALT)	32	IU/L	5.0 - 40.0
Total proteins	6.8	g/dL	6.6 - 8.7
Albumin	4.5	g/dL	3.40 - 5.00
Amylase	27.5	IU/L	40 - 125
Creatinine clearance	90	mL/min	>60
γ-GT (gamma-glutamyl transferase)	26	IU/L	5.0 - 36.0

**Figure 2:**
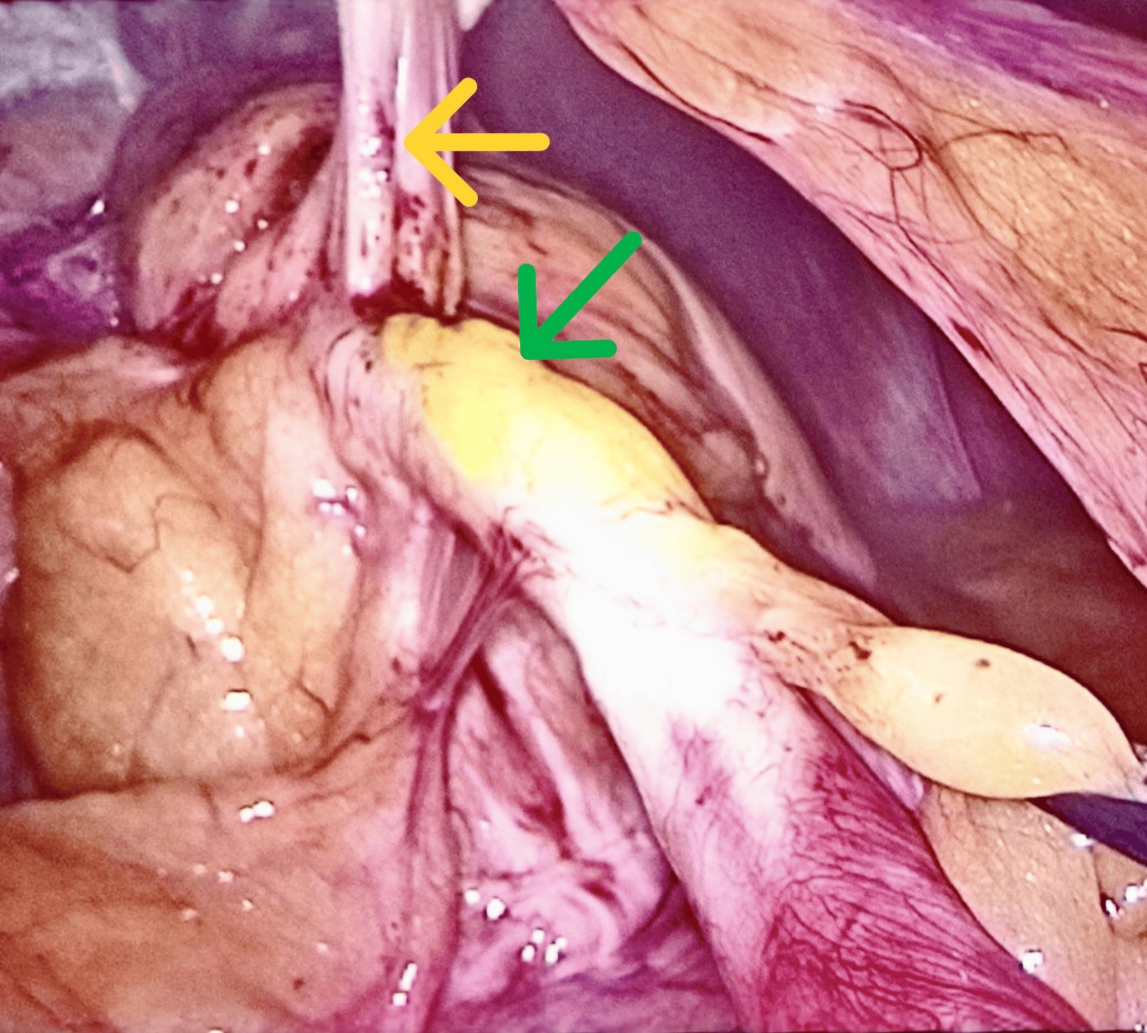
Symphysis (yellow arrow) and the tubing (green arrow) inside the small intestine

Thus, she was scheduled directly for laparoscopic removal of the gastric band. The procedure was performed under general anesthesia and with the patient placed in a modified dorsal lithotomy position. The laparoscopy revealed the gastric band positioned between the cardia and the lesser curvature, totally covered by fibrous tissue. The gastric tubing seemed to have entered the small intestine, having created a tunnel of approximately 20 cm in length inside the intestinal wall, exiting the bowel with a tightly attached symphysis between the intestinal helix and the abdominal wall (Figure 2).

**Figure 3:**
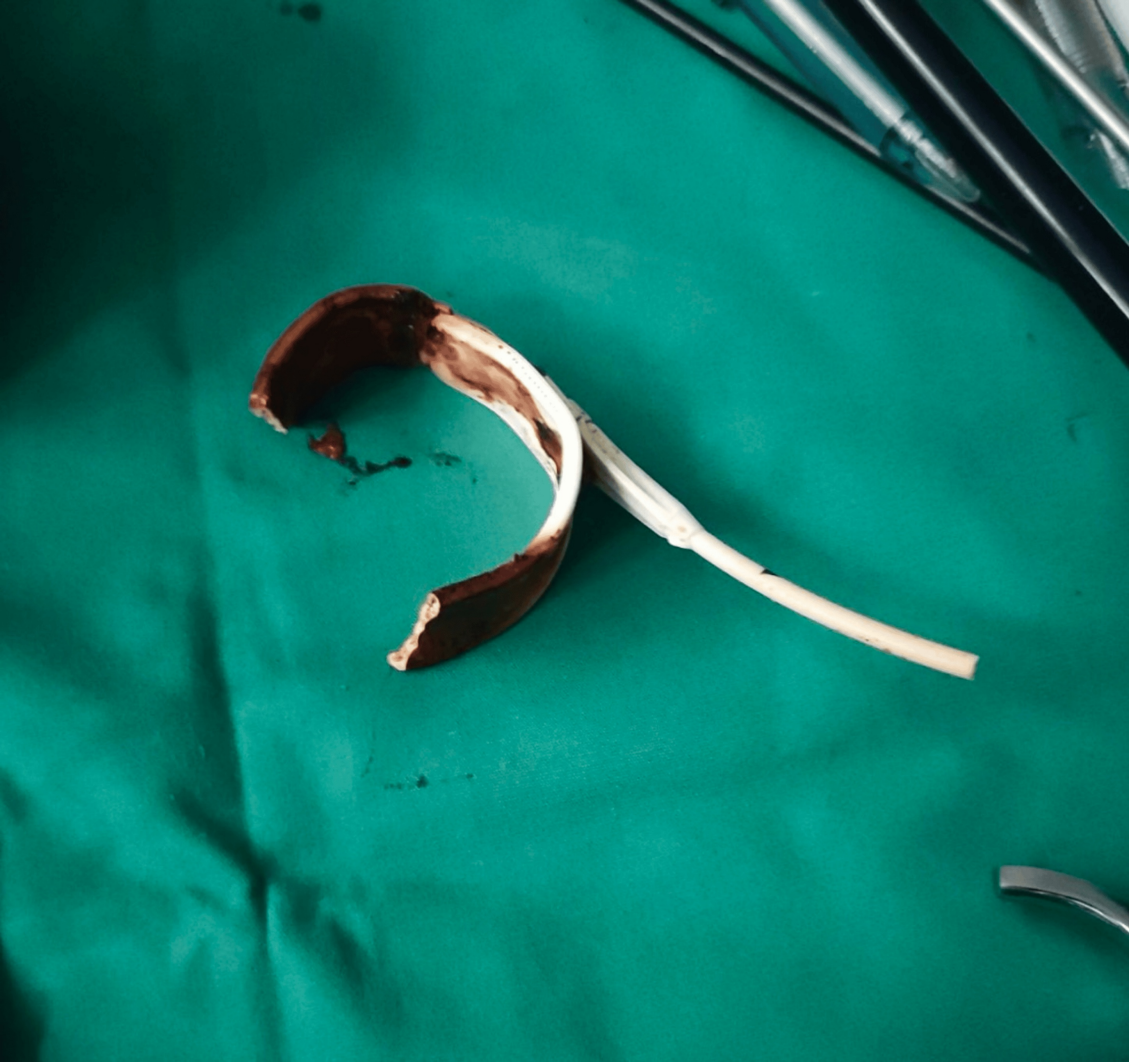
Gastric band that was removed together with a part of the tubing

It was impossible to recognize whether the tubing that was piercing the bowel was intraluminal. After complex symphysiolysis, the gastric tubing was cut, right at the point of entrance in the abdominal wall. Then the port, which protruded out of the skin, was removed together with a small part of the tubing. Afterward, the intestinal helix that contained the tubing was fully dissected and mobilized, and the fibrous tissue covering the gastric band was transected until the emergence of the buckle. An attempt was initially made to place a 12 mm intra-gastric trocar through the anterior gastric wall in order to continue the surgical procedure intra-gastrically; however, this was unsuccessful due to the gastric wall sliding over the trocar. Thus, the gastric incision was expanded and the gastric band was removed through the opening of the stomach (Figure 3).

The rest of the gastric tubing was removed from the intestinal tunnel as it was proven to be intraluminal. After suturing the stomach and the intestine at both ends of the tunnel, the device parts were removed, and the sutures were tested for leakage with the administration of methylene blue through the nasogastric tube. At last, after the laparoscopy was finished, the fibrous tissue covering the port was removed along with the skin and the defect was closed with a local advancement skin flap.

The immediate postoperative period was uneventful; however, the patient's hospitalization was extended due to a moderate episode of pulmonary embolism, and was discharged from our clinic on the 12th postoperative day. Based on the one-year follow-up, the patient is asymptomatic.

## Discussion

In the 1990s, laparoscopic adjustable gastric banding (LAGB) was considered the safest and least invasive procedure in bariatric surgery [9]. However, long-term experience with this technique showed an overall complication rate of up to 34% [9]. Thus, a significant decrease in its application has been observed. Some of the complications after LAGB are oesophageal dilatation, band erosion and migration, late slippages, and port problems [9]. In the retrospective study by Chisholm et al., conducted on 1874 patients who underwent LAGB, band erosion developed in 3.4% [10]. Out of the patients that developed this complication, 46% were asymptomatic, and the rest of them were symptomatic, with the most common symptoms being abdominal pain, obstruction, recurrent port infection, reflux symptoms, and sepsis [10]. Gastric band dislocation is a potentially serious complication that can be caused by factors such as over-tightening, improper placement, recognition of the band as a foreign body, and the patient’s behavior, such as high food intake. In a study that was conducted among 152 patients who had undergone LAGB, migration of the band was observed in 4.6% [11]. Patients with gastric band erosion and migration might present with nausea, abdominal pain, abdominal fullness, and weight gain. More severe complications, such as stomach ischemia, hematemesis, intestinal obstruction, intestinal perforation, and peritonitis, are infrequent [5,12]. However, these patients can also be asymptomatic and the dislocated band might be discovered by chance, when performing gastroscopy for other reasons, including for example epigastric pain or melena [13]. Three stages of gastric band migration have been described. In stage I a small part of the band is found in the gastric lumen. In stage II, more than half of the band is visible in the stomach, whereas in stage III, the band and the tube have completely migrated into the gastric lumen [9]. Cases of distal dislocation of the band have also been described, namely migration to the small bowel, causing intestinal obstruction or perforation, or even cases of migration to the rectum [12]. Regardless of the stage, the band has to be withdrawn in order to avoid further complications. Some authors suggest that if the patient is asymptomatic, it would be wise to wait until the complete migration of the band when the endoscopic removal would be easier [9]. Endoscopy and CT scan are the main diagnostic approaches in order to detect the dislocated band [12].

In our case, the patient was totally asymptomatic with the band fully migrated into the stomach, but with the atypical position of more than half the length of the tubing inside the lumen of an intestinal helix. The initial indication of surgery though, was the port erosion and protrusion through the skin which is clearly an alarm signal. The advances in minimally invasive techniques have allowed clinicians to manage the removal of the eroded and migrated band endoscopically, together with the extraction of the subcutaneous port through a separate small skin incision. This technique, avoids causing further damage to the gastric wall, already traumatized by the eroded band [1]. In particular, this is the treatment of choice when the gastric band has eroded more than 50% of the gastric wall. According to the available literature, when the band has eroded less than 33%, a stent is inserted and when it has eroded between 33-50%, a watch and wait strategy is preferable [13]. Moreover, if the migrated band is located after the level of the ileocecal valve, laparoscopy and mini-laparotomy might be indicated [8]. Rarely, as the band erodes the gastric wall and migrates into the small intestine, it might cause intestinal obstruction. In that case, Sleiman et al. presented a relatively non-invasive approach, namely the endoscopic removal using a pediatric colonoscope, which was proved to be easy, safe, and effective [6]. Biliary obstruction after LAGB has also been mentioned [14].

## Conclusions

Gastric band dislocation is a complex clinical entity that can present with various symptoms due to the different locations of the device migration and can cause potentially serious complications. Patient management varies depending on the symptoms, the band dislocation, and the performance status of the patient, but in general surgical or endoscopic management is considered. Subsequently, regular follow-up is crucial in patients who underwent bariatric surgery procedures. The management of gastric band dislocation depends on the severity of the condition and symptoms of the patient. Early intervention is essential to prevent further complications.

Our case highlights a rare complication because of the unusual device position between the stomach, the intestinal lumen, and the skin protrusion of the port, and the fact that the patient remained asymptomatic. Hence, we aim to underline the importance of a careful follow-up routine, even in asymptomatic patients and we encourage all patients who underwent bariatric surgery to adhere.
